# From diagnosis to dynamics: complex systems, transdiagnostics, and networks in forensic psychiatry

**DOI:** 10.3389/fpsyg.2026.1775635

**Published:** 2026-03-25

**Authors:** Stefan Bogaerts, Erik Masthoff, Iris Frowijn, Elien De Caluwé, Petra Habets, Marija Janković, Lee Smith

**Affiliations:** 1 Department of Developmental Psychology, School of Social and Behavioral Sciences, Tilburg University, Tilburg, Netherlands; 2 Fivoor Academy of Research, Innovation & Development (FARID), Rotterdam, Netherlands; 3 LIRA Lab, Department of Psychology, Faculty of Philosophy, University of Belgrade, Belgrade, Serbia; 4 Centre for Health, Performance and Wellbeing, Anglia Ruskin University, Cambridge, United Kingdom

**Keywords:** complexity theory, forensic - psychiatric practice, forensic psychiatry, network analysis, transdiagnostic approach

## Abstract

**Introduction:**

Forensic psychiatry is characterized by highly complex case presentations, where co-morbidity, fluctuating symptoms, and elevated risk of disruptive behavior are central concerns. Traditional categorical diagnostic models, such as the Diagnostic and Statistical Manual of Mental Disorders, Fifth Edition (DSM-5), often fail to capture this clinical complexity. This article introduces an integrated framework that synthesizes three perspectives: complexity theory, transdiagnostic models, and network analysis.

**Methods:**

Using a hypothetical yet realistically constructed case (Mark), we demonstrate how these approaches can collectively lead to a richer clinical conceptualization and a more effective treatment strategy.

**Results:**

Complexity theory offers a dynamic lens through which symptoms are understood as emergent patterns within a feedback-sensitive system. The transdiagnostic approach shifts the focus toward cross-cutting psychological processes, such as emotion regulation and impulsivity, that often constitute the core of psychopathological issues. Network analysis further elucidates the interrelations between symptoms and processes and highlights central or “bridge” symptoms as potential focal points for intervention. Two conceptual network models of Mark’s symptoms and underlying mechanisms illustrate how this integrated approach can enhance both diagnostic clarity and therapeutic precision. Instead of a fragmented treatment plan based on multiple discrete diagnoses, a coherent systemic perspective is established, guiding prioritization and clinical monitoring.

**Discussion:**

The discussion addresses the added value, practical applications, ethical considerations, and implementation challenges of this approach, particularly in forensic psychiatric contexts. This integrated conceptualization holds promise for renewing forensic diagnostics and treatment paradigms, shifting from static disorder classification to dynamic, person-centered networks.

## 1 Introduction

Forensic psychiatry is characterized by complex interactions of severe and diverse psychopathology and elevated risk of disruptive or violent behavior. Patients in this field frequently present with multiple diagnoses and fluctuating symptoms, making traditional diagnostic systems, such as the Diagnostic and Statistical Manual of Mental Disorders, Fifth Edition (DSM-5; American Psychological Association (APA), 2013) less suitable. Categorical systems often fail to adequately capture the dynamic and comorbid nature of such clinical presentations (American Psychological Association (APA), 2013; Widiger and Samuel, 2005). For instance, an individual may simultaneously exhibit features of depression, trauma-related disorders, and impulse control problems, making it difficult for any single diagnostic label to reflect the full clinical picture (Mullins-Sweatt et al., 2020). Empirical studies confirm that forensic psychiatric patients frequently suffer from multiple co-occurring disorders: high prevalence rates of personality disorders (50–90%), mood disorders (20–60%), and psychotic disorders (15–20%) are often accompanied by substance abuse (Jeandarme et al., 2017; Bogaerts et al., 2020). This leads to fragmented diagnostics and complicates the formulation of a coherent treatment plan.

In response to these limitations, integrated perspectives are gaining traction within mental healthcare. This manuscript illustrates, through the hypothetical case study of “Mark^1^,” how three conceptual approaches, namely complexity theory, transdiagnostic models, and network analysis, can be combined into an alternative framework for diagnosis and intervention. Rather than viewing psychopathology as a collection of distinct disorders, the integrated approach conceptualizes it as a dynamic interaction of underlying processes and symptoms. Each approach provides a limited perspective on the complexity of forensic psychopathology. Diagnostic models classify symptoms but tend to remain largely static. Transdiagnostic models identify shared mechanisms across disorders, although these often stay abstract. Network analysis visualizes symptom interactions but risks becoming merely descriptive without a guiding framework. Used in isolation, each leaves important gaps. By integrating these approaches, we link dynamic change, shared mechanisms, and concrete symptom interactions, improving both explanatory power and clinical interpretability in a way that none of them can achieve alone.

We begin by outlining the theoretical background of these three approaches and their interrelationships. We then present Mark’s case and analyze his symptomatology using conceptual network models. Finally, we explore the clinical implications of this integrated perspective, with special attention to its added value in clinical decision-making and treatment planning within forensic psychiatric contexts. This setting is particularly well-suited for such an approach, given the high rates of co-morbidity, severe behavioral disturbances, and dynamic risk factors inherent to this population (Howard et al., 2013; Jeandarme et al., 2016). Clinicians need models that help them structure this complexity, monitor risk development over time, and intervene effectively at critical moments. The integration of complex systems thinking, transdiagnostic reasoning, and network-based modeling offers a powerful framework for meeting these needs (Nelson et al., 2017).

### 1.1 Theoretical framework

#### 1.1.1 Complex systems approach

Complexity theory views mental disorders not as fixed entities located within individuals, but as phenomena emerging from dynamic interactions within a broader system. Instead of defining disorders as stable, discrete entities, this approach emphasizes variable patterns of behavior and experiences that emerge from the continuous interplay of their constituent components. Here, the term complex systems approach is used to denote the application of principles from complexity theory (e.g., non-linearity, emergence, feedback loops) to psychopathology, rather than complexity theory as a formal mathematical discipline. In this view, psychopathology is not a static “disease” that an individual possesses, but rather the outcome of a constantly evolving network of interacting factors, biological, psychological, and environmental (Robinaugh et al., 2020). These patterns are understood as emergent properties of the system. Consequently, small perturbations in a single element may sometimes lead to disproportionately large consequences at the system level, akin to the “butterfly effect.” Mental disorders are thus seen as causal systems composed of mutually reinforcing symptoms. Symptoms and risk factors are embedded in feedback loops, giving rise to non-linear dynamics (McNally, 2021). Positive feedback mechanisms, for instance, can trigger cascading effects, creating self-perpetuating vicious cycles. A well-documented example is “critical slowing down” in mood states (van de Leemput et al., 2014). As an individual approaches the onset of a depressive episode, subtle dynamic changes intensify; emotional states become more autocorrelated (they persist longer), and emotional variability increases. These early warning signals indicate that the system is nearing a tipping point. Once this threshold is crossed, the system collapses into a new, more stable state, such as a full depressive episode (e.g., Olthof et al., 2019).

These theoretical insights have direct clinical implications. Insights from complexity science thus highlight the importance of temporal dynamics and early warning indicators: monitoring subtle shifts in mental states can aid in early detection of relapses or crises (Ongur and Paulus, 2025). Van de Leemput et al. (2014) demonstrated, for example, that in depressed patients, a transition toward recovery or relapse is typically preceded by increased emotional variability and a slowed return to baseline, hallmarks of an unstable system on the verge of transformation. Complex adaptive systems may also undergo phase transitions: a seemingly stable pattern can suddenly shift due to the accumulation of small changes. This is particularly relevant in forensic psychiatry. For example, rising insomnia or irritability may gradually escalate into an aggressive outburst once a critical threshold is reached (Nelson and Trainor, 2007).

Traditional diagnostic frameworks struggle to accommodate such dynamic processes. In contrast, the complex systems approach provides a framework for understanding not only the presence of symptoms, but also their mutual interactions and temporal fluctuations. From this perspective, clinicians are encouraged to monitor systemic indicators, such as sleep patterns, mood variability, and stress levels, as potential precursors of decompensation or shifts in functioning (Bant and Bogaerts, 2024).

#### 1.1.2 Transdiagnostic approach

The transdiagnostic perspective shifts the focus from diagnostic labels to the underlying processes that are common across multiple mental disorders. Many psychological conditions share core mechanisms such as maladaptive emotion regulation, impulsivity, or negative thinking patterns, which are not confined to any single DSM category (Harvey et al., 2004). Rather than treating patients strictly according to disorder-specific protocols, the transdiagnostic approach advocates for interventions that target these overarching mechanisms that sustain distress across diagnostic boundaries.

A classic example is emotion dysregulation, which plays a central role in disorders such as borderline personality disorder, post-traumatic stress disorder (PTSD), depression, and anxiety disorders (Kunst et al., 2011). Similarly, impulsivity is observed in substance use disorders, antisocial personality disorder, and ADHD, while rumination is relevant across both anxiety and mood disorders. Harvey et al. (2004) introduced the idea that many mental disorders are maintained by shared cognitive-behavioral processes, including attentional biases, memory distortions, and interpretative tendencies.

More recent research has expanded and refined this list. For example, Wade et al. (2025) identified 38 unique transdiagnostic processes that function as risk or maintenance factors across various forms of psychopathology. A transdiagnostic process is defined as a mechanism that is present across multiple disorders and contributes to their onset or persistence. Examples include heightened anxiety sensitivity, perfectionism, rumination, and neurobiological vulnerabilities such as sleep deprivation and stress reactivity (e.g., Smith and Alloy, 2009). In addition to identifying these processes, some studies within the transdiagnostic approach use latent variable methods (e.g., factor analysis or item response theory) to uncover the underlying constructs linking different symptom expressions, which conceptually differ from but complement the network analytic approach described below.

The primary advantage of the transdiagnostic approach is that it enables more integrated and accessible treatment planning. Instead of pursuing a separate treatment trajectory for each comorbid diagnosis, a single transdiagnostic protocol can simultaneously address multiple areas of dysfunction. Some evidence-based therapies already target such broad mechanisms. For instance, Barlow et al. (2016) Unified Protocol was developed to treat emotional disorders transdiagnostically by focusing on emotion regulation skills, cognitive flexibility, and related domains. Similarly, Dialectical Behavior Therapy (DBT), originally designed for borderline personality disorder, is now recognized as a transdiagnostic intervention for emotion regulation difficulties and impulsive, self-destructive behavior across diagnoses (e.g., Neacsiu et al., 2010).

In Mark’s case, instead of treating his PTSD, depression, and personality pathology as separate entities, it may be more effective to target shared dysfunctions such as chronic emotion dysregulation, impulsivity, and interpersonal distrust. This approach facilitates a more cohesive and personalized treatment plan that aligns closely with his clinical needs. Research suggests that such a process-focused approach may not only be more efficient but also potentially more effective for patients with co-morbid dysfunctions, as it addresses the root mechanisms of distress rather than treating symptoms piecemeal by diagnosis (van der Heijden et al., 2020; Widiger, 2021).

Beyond treatment implications, the transdiagnostic framework also offers a novel conceptual lens for diagnosis. Initiatives like the Hierarchical Taxonomy of Psychopathology (HiTOP) classify mental disorders along dimensional spectra, departing from rigid categorical classifications (Kotov et al., 2017). In this model, disorders are reorganized based on shared features, forming broad spectra. The internalizing spectrum refers to problems that are primarily directed inward, such as fear and distress, while the externalizing spectrum involves outwardly directed behaviors, such as antisocial behavior, substance use, and impulse control problems. These spectra are underpinned by transdiagnostic traits such as negative affect (which underlies both anxiety and depression) and disinhibition (which contributes to impulsive and antisocial behavior). Such models aim to more accurately reflect the overlap commonly seen in clinical practice by organizing symptoms along continuous dimensions and shared underlying liabilities, rather than imposing rigid diagnostic thresholds. Zárate-Guerrero et al. (2022) argue that current DSM categories often impose artificial boundaries, despite significant symptomatic overlaps, especially between anxiety and depression, which are frequently better treated as interconnected phenomena. Overall, the transdiagnostic perspective enables clinicians to reframe Mark’s problems not as a checklist of disorders, but in terms of core underlying processes, such as emotion regulation, impulse control, and trust in others, leading to a more integrated and clinically meaningful formulation. A transdiagnostic perspective may impact medication prescribing by targeting shared mechanisms like emotion dysregulation, rather than separate diagnoses. Using treatments such as mood stabilizers could reduce polypharmacy by addressing core processes affecting multiple symptoms. While promising (Insel, 2014), this approach requires further research and clinical caution, as current prescribing guidelines largely remain rooted in diagnosis-specific frameworks.

#### 1.1.3 Network analysis

The network approach to psychopathology serves both as a theoretical framework and as an analytical methodology. Central to this perspective is the notion that symptoms are not merely passive reflections of an underlying disorder, but instead actively influence and sustain one another (Bogaerts et al., 2021). A disorder, in this model, is conceptualized as a network of causal interactions among symptoms. For instance: insomnia → reduced concentration → work-related problems → depressed mood → insomnia, and so on (Borsboom and Cramer, 2013; Bant and Bogaerts, 2024). In such a symptom network, symptoms are represented as nodes, and their connections, or edges, depict statistical or hypothesized relationships and interactions, indicating how symptoms may influence or co-occur with one another. Network analysis makes this structure explicitly visible and quantifiable. Importantly, some symptoms may play a more central role within the network than others. Measures of centrality (e.g., degree, betweenness) identify which nodes are highly connected within the system (Bogaerts et al., 2020). Central nodes, or in this paper, central symptoms, are strongly connected to other symptoms, such that changes in these nodes can affect multiple related symptoms. Highly central symptoms represent the most influential nodes, acting as primary drivers and promising targets for intervention, as changes in these nodes may ripple through the network and induce broader improvements.

A bridge symptom, in contrast, is specifically a node that links distinct symptom clusters or diagnostic categories, allowing changes in the node to affect multiple domains. It can help explain the development and maintenance of co-morbidity between mental disorders: when symptom overlap occurs, one condition may trigger or sustain the other through a bridging symptom (Heeren et al., 2018). Fried and Cramer (2017), for example, argued that bridge symptoms, strongly associated with multiple disorders, can increase the risk of developing additional mental health conditions. In depression-anxiety networks, insomnia and concentration problems are frequently identified as bridge symptoms connecting the two syndromes. Because of their linking role, both central and bridge symptoms constitute particularly important targets, as effectively addressing them can “pull” the system toward improvement.

Network analysis also offers new methodological possibilities. Using statistical techniques such as graphical LASSO (Tibshirani, 1996), one can extract a symptom network from data that includes only the most likely and robust connections. Longitudinal network models are increasingly being developed, in which time series data, often collected via Ecological Momentary Assessment (EMA), are used to map person-specific networks. It is essential to distinguish between static (cross-sectional) and dynamic (time-series) networks. While static networks analyze symptoms at a single time point, dynamic networks capture how symptoms influence each other over time through repeated measurements (Nemani et al., 2025). This allows clinicians and researchers to investigate not only the structure but also the temporal dynamics and directionality of symptom interactions.

This is particularly valuable in forensic settings, where crises may escalate rapidly and unpredictably. A dynamic network model can reveal how an individual’s symptoms interact on a day-to-day basis. This aligns closely with the complex systems approach by illustrating feedback loops and signaling potential tipping points. For example, Bringmann et al. (2013) found that network connectivity increased in some depressed patients prior to relapse, indicating reduced psychological resilience, a phenomenon known as critical slowing down.

In clinical practice, a network diagram can serve as a visual tool to clarify case conceptualization. It immediately reveals which symptoms are tightly linked and highlights potential bottlenecks or therapeutic leverage points. The network perspective enriches traditional diagnostics by attending to interaction effects, not just which symptoms a person has, but how these symptoms co-occur and influence each other.

In the case of Mark, for example, a network model might show that anger, impulsivity, and alcohol use form a tightly interconnected cluster that contributes directly to violent incidents, something less apparent when viewed through separate DSM diagnoses such as PTSD or depression (Bant and Bogaerts, 2024). Network analysis can also be used to monitor whether a reduction in one symptom (e.g., insomnia) corresponds with decreases in related symptoms (e.g., irritability, aggression), thereby helping to evaluate treatment progress. All in all, network analysis bridges the gap between theory and practice. It compels clinicians to formulate explicit hypotheses about causal symptom relationships and makes these transparent and actionable for both practitioner and patient alike (e.g., Wade et al., 2025).

### 1.2 Integration of perspectives

Although each of the approaches offers a distinct perspective on psychopathology, they are complementary. A unifying feature is their rejection of reductionism and simplistic one-to-one mappings between disorders and symptoms. Instead, these models conceptualize psychological difficulties as arising from multiple, interacting factors. The complex systems approach provides the overarching dynamic framework in which non-linear interactions and emergent phenomena take center stage. The transdiagnostic perspective adds that these interactions often revolve around processes that cut across diagnostic boundaries, bridging seemingly disparate symptoms through shared underlying mechanisms. Finally, network analysis offers tools to concretely map and analyze these intricate and transdiagnostic relationships (e.g., Barlow et al., 2016; Wade et al., 2025).

Together, these approaches form an integrated perspective that reframes Mark’s clinical presentation, not as a set of discrete diagnoses, but as a single, interconnected system. This system encompasses both his symptoms (e.g., anger, sadness, insomnia) and the underlying processes (e.g., impulsivity, distrust, trauma processing), showing how these elements mutually influence one another. Such a model can provide clinical traction exactly where traditional methods fall short. For instance, this integrated view helps explain why Mark might resort to alcohol following a trauma-related flashback. By combining transdiagnostic insights (such as difficulties in emotion regulation) with network analysis that maps the link between trauma triggers and substance use, the causal pathway becomes explicit. The overarching aim is to arrive at a more realistic and actionable conceptualization of complex clinical presentations, one that directly informs intervention strategies. Importantly, these approaches not only coexist but also inform one another. Complexity theory provides dynamic logic, transdiagnostic models identify key processes, and network analysis translates these insights into a clinically actionable structure. Without this integration, clinicians risk staying at the level of abstract theory or working with detailed but unguided symptom maps.

In the sections that follow, we illustrate this integrated approach using the case of Mark. We begin by outlining the methodological framework and providing background information on Mark. Subsequently, we present conceptual network models that visualize his symptoms and their interrelationships.

## 2 Method

### 2.1 Analytical framework for case illustration

This paper provides a conceptual exploration of integrating complexity theory, transdiagnostic models, and network thinking into forensic psychiatric case formulation. Both the case and network models are illustrative and theory-informed, rather than empirically derived. The case of Mark is a hypothetical patient, constructed based on existing forensic psychiatric case material and relevant literature. His network models were developed through expert estimation and hypothesis generation, guided by current scientific insights. Specifically, theories on symptom connectivity and transdiagnostic processes (e.g., Bringmann et al., 2022) were used to postulate plausible associations among Mark’s difficulties. Network visualizations were created using Gephi (Bastian et al., 2009), an open-source software platform for network visualization. This approach aligns with the aim of this paper: to explore how an integrated model of complexity theory, transdiagnostics, and network thinking can be applied in clinical practice. Mark was selected because his profile reflects a commonly observed forensic pattern characterized by childhood trauma, comorbid symptoms, substance use, and elevated risk of violence. Although no single case can be fully representative, Mark illustrates a pattern frequently encountered in forensic psychiatric practice. Cases of this nature are inherently multifaceted, often demanding integrative and innovative assessment and intervention strategies. The presented frameworks can facilitate a nuanced understanding and formulation of such clinical profiles.

**Figure 1:**
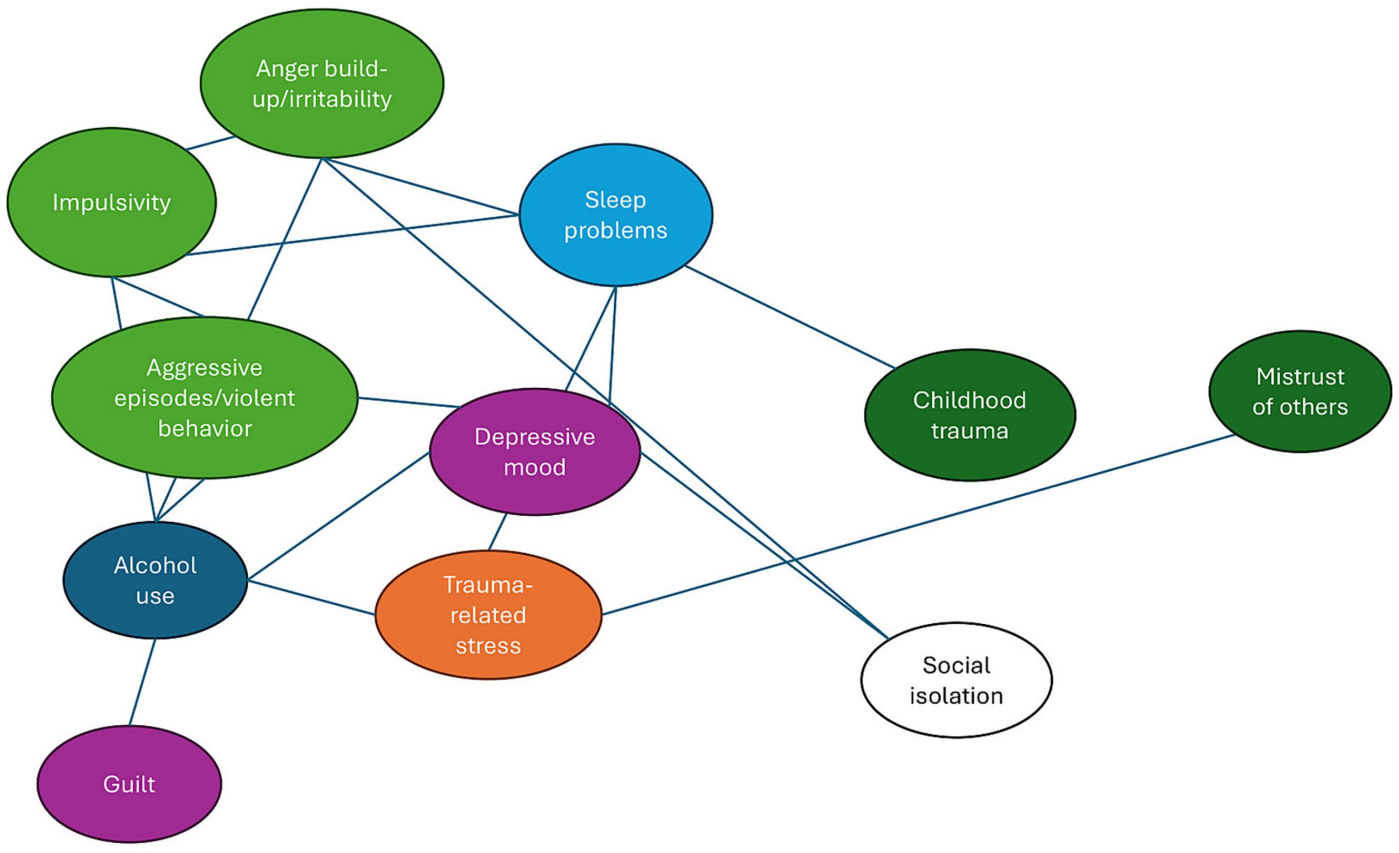
Mark’s psychopathology network. This network is a conceptual visualization based on expert knowledge and literature; it is not derived from empirical data and is intended for clinical hypothesis generation. Connections are undirected (no arrows) and unweighted, to emphasize mutual influences rather than causal strength or direction. Colors are used to group related symptoms and processes for readability and do not imply fixed or standardized meanings across figures.

**Figure 2:**
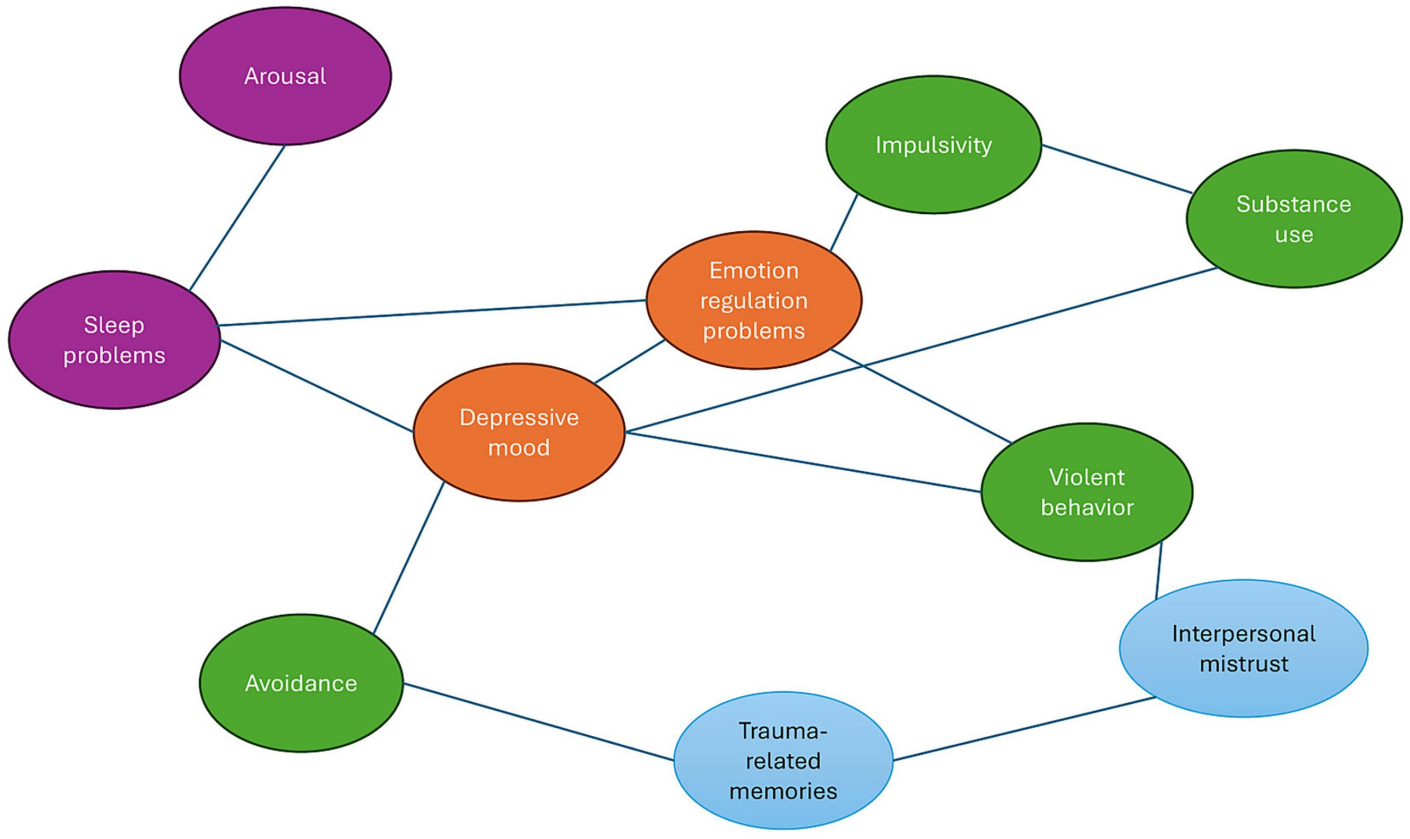
Mark’s transdiagnostic process network. This network is a conceptual visualization based on expert knowledge and literature; it is not derived from empirical data and is intended for clinical hypothesis generation. Connections are undirected (no arrows) and unweighted, reflecting bidirectional relationships without implying direction or magnitude. The network depicts selected, theoretically relevant connections discussed in the text, rather than an exhaustive representation of all possible relationships. Colors are used for visual clarity and do not correspond to the color coding used in Figure 1, nor do they carry inherent clinical meaning.

Two conceptual network models were constructed. The first is a complex systems network that groups Mark’s core symptoms into thematic clusters and depicts hypothesized interactions among them (Figure 1). The second is a transdiagnostic symptom network that visualizes the key transdiagnostic processes in Mark’s case and their interrelations (Figure 2). These models are grounded in theoretical literature. For example, it is hypothesized that alcohol use functions as a bridge between impulsivity and mood-related complaints, or that sleep disturbances serve as both early indicators and causal contributors to irritability and trauma-related re-experiencing (e.g., Green et al., 2023; Werner et al., 2024). In building these models, we deliberately identified symptom clusters to make the complex system more interpretable for clinical purposes.

We opted for undirected network models, meaning that the connections between nodes are not depicted with arrows. This decision was based on four considerations: First, the visualizations are conceptual and exploratory, not based on empirical time series or causal data. Second, this approach highlights the bidirectional influence between symptoms and processes, consistent with common patterns in psychopathology (i.e., feedback loops). Third, this approach aligns with systems thinking, in which emergent behavior arises from interactions without fixed directional causality. Fourth, undirected models enable a holistic view of the system without speculative or uncertain assumptions about the direction of influence. In future iterations, informed by longitudinal data or experimental interventions, the model could be adapted into a directed network. For the present purposes, however, the undirected model provides a robust and transparent foundation for case conceptualization. The network models are presented below, with an accompanying explanation for each figure. We note that these models have not been empirically validated; they are intended to plausibly illustrate insights achievable through an integrated case formulation. Accordingly, the network figures should be understood as clinical thinking tools that support hypothesis generation and discussion, rather than as precise or definitive maps of psychopathology.

### 2.2 Case description: Mark

Mark is a 35-year-old man admitted to a Forensic Psychiatric Center following a violent offense committed under the influence of alcohol. He grew up in an unstable family environment marked by repeated episodes of physical and emotional abuse, constituting severe childhood trauma. During adolescence, signs of emotional dysregulation and impulsive behavior started to appear. Over time, Mark developed a pattern of substance use, primarily alcohol and amphetamines, presumably as a form of self-medication to soothe internal unrest. His psychiatric history is notably complex. He exhibits symptoms consistent with multiple disorders: intense mood swings, chronic feelings of emptiness, and fear of abandonment (characteristic of borderline personality disorder); intrusive nightmares and hypervigilance (indicative of PTSD); and recurring depressive episodes marked by guilt and worthlessness. Over recent years, Mark has received comorbid diagnoses of PTSD, borderline personality disorder, alcohol use disorder, and recurrent depression. However, these individual labels fail to fully explain the nature of his behavior, in part due to significant symptom overlapping. Interpersonally, Mark functions in a disorganized manner. His relationships are characterized by a combination of extreme dependency and profound mistrust. At times, he is dependent and jealous of others, distant and suspicious. During therapy sessions, Mark reports experiencing “blackouts” during emotionally intense conflicts, during which he later recalls little of his aggressive outbursts. He describes a sense of losing control: feeling as though a minor trigger can suddenly make him “lose control.” These impulsive episodes are particularly likely to occur under the influence of alcohol, which further diminishes his self-restraint. Mark has been involved in multiple violent incidents, ranging from physical altercations to, in the index offense, the severe assault of a housemate. After such events, he typically experiences deep remorse and shame, which in turn exacerbates his depressive symptoms. This leads to avoidance behaviors, withdrawal from social contact, and often a return to alcohol use to mitigate his feelings of guilt, perpetuating a vicious cycle.

In summary, Mark struggles with an interconnected cluster of issues: trauma-related symptoms, affective instability, difficulties with impulse and aggression regulation, substance use problems, and interpersonal dysfunction. Traditional diagnostic frameworks classify these as distinct co-occurring problems, but they offer no clear explanation for why these issues are so tightly interwoven in Mark’s case. In the following sections, we aim to provide an explanatory framework and visual representation of the interplay among Mark’s symptoms using complex network analysis and transdiagnostic reasoning.

## 3 Results

### 3.1 Network modeling of mark’s psychopathology

Before presenting the network models, we first articulate why traditional diagnostic and treatment approaches are challenging in Mark’s case. The DSM classification system applied labels such as “borderline” and “PTSD” but failed to account for the interactions among his various issues. As a result, his treatment plan became fragmented: separate protocols were applied for trauma and for aggression regulation, with little integration between them. Crucial risk factors were viewed in isolation, even though in reality they reinforced one another, e.g., trauma-related stress → insomnia → irritability → aggression.

Mark’s clinical profile illustrates the complex overlap and dynamic interplay that conventional DSM categories cannot adequately address. As Hamaker (2012) argued, it is essential to attend to within-person variability and feedback loops, since symptoms can mutually reinforce or weaken each other over time. In Mark’s case, each substance use relapse was not an isolated incident but was precipitated by an emotional build-up (e.g., anger, tension), which was further intensified by insomnia and trauma triggers. This interconnectedness remained largely invisible within conventional diagnostic approaches and contributed to insufficiently coordinated treatment efforts.

### 3.2 Network visualization of mark’s symptoms

To clarify the complex constellation of Mark’s complaints, we constructed a conceptual systems network (Figure 1).

The network in Figure 1 illustrates hypothesized interactions between Mark’s core symptoms, grouped into thematic clusters. Colors represent distinct domains: dark green (childhood trauma and mistrust), orange (trauma-related stress), green (impulsivity and anger build-up), purple (depressive mood and guilt), light blue (sleep problems), white (social isolation), and dark blue (alcohol use). Lines indicate hypothesized links; nodes with more connections (e.g., alcohol use, sleep problems) represent central or bridging factors that may have a cascading impact on other factors. Within each cluster, symptoms are interconnected. For example, childhood trauma is closely linked to trauma-related stress, which in turn fuels persistent mistrust of others. Impulsivity and anger build-up are also closely associated and characterize Mark’s aggressive episodes. Alcohol use is closely linked to this cluster: it amplifies impulsive outbursts and directly contributes to violent acts, while also being triggered in reverse by feelings of guilt. These guilt dynamics are reflected as purple clusters. After an aggressive incident, Mark experiences depression, which leads to social isolation and alcohol use. Sleep problems occupy a somewhat separate position but are connected to multiple clusters, such as childhood trauma, impulsivity, and social isolation. Chronic insomnia depletes Mark’s energy, heightens trauma-related stress, and worsens depressive symptoms. Social isolation stems from childhood trauma and irritability.

Several key bridge symptoms stand out in this network. Alcohol use links the anger build-up cluster with the depressive mood cluster, functioning as a catalyst for cascading effects. According to the model, reducing alcohol use (e.g., through treatment) could decrease both Mark’s aggressive outbursts and his depressive mood and guilt, creating a positive chain reaction. Sleep problems connect childhood trauma and irritability to mood-related complaints. The literature suggests that such symptoms, especially sleep and energy disturbances, often have cross-diagnostic effects (van Veen et al., 2021). In Mark’s case, improving sleep could potentially yield dual benefits: reducing both irritability and depressive burden. As shown in Figure 1, Mark’s violent behavior stems not just from “antisocial traits,” but from the combined effects of trauma, heightened stress sensitivity, poor coping strategies (alcohol), and insufficient regulatory skills. From a clinical perspective, this constellation of factors is well-recognized. In routine practice, patients with presentations similar to Mark are often perceived as unpredictable or challenging, not due to any single trait, but because minor stressors can activate a cascade of reactions that may overwhelm both patient and clinician. This complex-dynamic perspective aligns with empirical findings suggesting that violent offenses typically result from the interaction of multiple, reinforcing risk factors (e.g., Bogaerts et al., 2020; Bant and Bogaerts, 2024).

For the clinician, this model offers practical utility: it allows for targeted identification of central or strongly connected links in the network, namely connections whose disruption is most likely to produce widespread change. For instance, the network suggests that addressing alcohol use is highly strategic, given its centrality and high connectivity. This supports the network hypothesis that intervention on central nodes can trigger a cascade of improvement throughout the system (e.g., Bogaerts et al., 2020). Clinically, this perspective informs treatment sequencing. In Mark’s case, the network suggests that early focus on alcohol use or sleep regulation may be more effective in reducing risk and emotional instability than immediately targeting trauma-related content.

### 3.3 Visualization of transdiagnostic processes

Figure 2 depicts the underlying transdiagnostic processes in Mark’s case. These processes are color-coded by type: light blue (cognitive processes), orange (emotional processes), light green (behavioral processes), and violet (physiological processes). The network illustrates how central mechanisms, such as emotion regulation problems and impulsivity, are closely interconnected with substance use and depressive mood.

This conceptual model shows the main transdiagnostic processes underlying Mark’s difficulties. This model includes nodes representing emotion regulation problems, impulsivity, interpersonal mistrust, trauma-related memories, substance use, violent behavior, sleep disturbances, arousal, depressive mood, and avoidance. The connecting lines indicate hypothesized direct influences between these factors. For example, Figure 2 shows that emotion regulation difficulties (orange) are directly connected to impulsivity and violent behavior (green nodes). Mark’s inability to manage intense emotions leads to impulsive reactions and aggressive outbursts. Impulsivity, in turn, is associated with substance use: his tendency toward immediate gratification increases the likelihood of alcohol and drug use. Trauma-related memories (blue) appear as a key node as well, linked to both interpersonal mistrust and avoidance, two hallmark features of PTSD. In Mark’s case, these traumatic memories contribute to distrust of others and conflict avoidance. This mistrust is also linked to violent behavior, likely because Mark tends to perceive hostility in social situations and responds with aggression. A paranoid interpretation (“they’re out to get me”) can escalate into a preemptive attack. Depressive mood (orange) is connected to both avoidance and substance use: when experiencing low mood, Mark withdraws and turns to alcohol for comfort. This pattern creates a vicious cycle, as substance use ultimately worsens its depression through both health and social consequences. Sleep problems (violet) emerge as another prominent factor in the network. These are connected to both emotion regulation difficulties and depressive mood. Sleep deprivation undermines Mark’s emotional resilience and intensifies his regulation problems, a known transdiagnostic effect of insomnia across mood and anxiety disorders. Physiological states such as sleep deprivation and heightened arousal also function as bridge nodes in this network. These psychophysiological factors influence both emotion regulation and impulsivity and can serve as early predictors of escalation. Including these variables underscores the importance of biological monitoring (e.g., heart rate variability, sleep tracking) as potential early warning signals (de Looff et al., 2019; Jankovic et al., 2021). Additionally, violent behavior (green) is directly linked to depressive mood. Following an act of violence, Mark experiences guilt and low mood. This illustrates how external behavior (violence) feeds back into internal states (depression) through moral emotion (shame).

### 3.4 Interpretation of the transdiagnostic process network

The transdiagnostic network highlights which core processes are central to Mark’s difficulties. Emotion regulation problems, impulsivity, and interpersonal mistrust stand out as highly connected nodes, consistent with the hypothesis that these are key intervention targets. This supports a treatment approach that combines Dialectical Behavior Therapy (focusing on emotion regulation and impulse control) with schema therapy or trauma-focused therapy (addressing mistrust and trauma processing). Furthermore, Figure 2 reinforces the role of substance use as a pivotal factor in the maintenance of multiple issues, from aggression to depression. This aligns with forensic research indicating that alcohol and drug use are significant risk factors for violent recidivism among comorbid patients (Bogaerts et al., 2018).

### 3.5 Integrated perspective on transdiagnostic processes and networks

Figures 1 and 2 demonstrate that Mark’s symptom clusters and underlying processes are deeply interconnected. These complementary networks not only identify which problems are present but also clarify how they influence one another across different domains. This integrated perspective reveals that improvement in one domain will likely affect others. For instance, successful treatment of Mark’s alcohol dependence may reduce his risk of aggression while simultaneously improving sleep and mood, given alcohol’s central role in both figures. Conversely, neglecting his insomnia may perpetuate emotional instability and increase the risk of relapse into substance use. These reciprocal dynamics underscore the necessity of a coordinated, broad-spectrum treatment strategy, rather than fragmented interventions targeting isolated problems. Such an integrated network view provides clinicians with a roadmap for prioritizing interventions that can produce the largest cascading effects.

## 4 Discussion

The case of Mark illustrates how the integration of a complex systems approach, transdiagnostic processes, and network modeling can yield a richer and more nuanced clinical understanding than traditional diagnostic approaches alone. In this section, we evaluate the benefits and limitations and explore its implications for clinical decision-making in forensic psychiatry.

### 4.1 Added value of the integrated approach

#### 4.1.1 Holistic view of the patient

Rather than treating diagnoses as isolated silos, this approach allows us to view Mark as a unified system in which symptoms and underlying processes are deeply interconnected. This results in more comprehensive and strategically targeted interventions (Borsboom et al., 2019). For example, Mark’s network suggests that addressing a central issue, such as substance use, may have cascading effects on related symptoms like aggression and mood. This aligns with the principle of network intervention. By addressing a central or bridging symptom, clinicians may initiate broader system-wide improvements. Robinaugh et al. (2020) emphasized that identifying such key nodes is crucial for sequential, symptom-specific treatment to be effective. The construction of these network models typically begins with a thorough clinical intake, in which not only symptoms but also behavioral patterns, contextual triggers, and coping strategies are explored. Structured clinical interviews, Routine Outcome Monitoring (ROM), and EMA can serve as valuable sources of input for identifying the traits and behaviors that populate the networks.

#### 4.1.2 Focus on mechanisms instead of labels

The transdiagnostic perspective helps prevent the oversight of crucial psychological processes that do not fit neatly within DSM categories. In Mark’s case, a disorder-specific view might not prioritize emotion regulation as a treatment focus, since it is not a stand-alone diagnosis. Yet from a transdiagnostic standpoint, emotion dysregulation underlies many of his symptoms. Figure 2 supports this, showing emotion regulation as a central hub linked to impulsivity, violence, and depression. The integrated model thus prioritizes underlying mechanisms over diagnostic labels, which may have implications for access to care, reimbursement and clinical decision-making (van der Heijden et al., 2020). This means tailoring treatment to individual needs rather than following one-size-fits-all protocols. At the same time, transdiagnostic and skills-based interventions are not equally effective for all patients. Some individuals may initially lack the stability, motivation, or reflective capacity required for such approaches, underscoring the need for careful clinical judgment and flexibility in treatment planning. For example, for those with intellectual disabilities or acquired brain injury, treatment may rely more heavily on structured behavioral support or environmental interventions, such as clear routines, stepwise guidance, prompts, and reinforcement strategies.

#### 4.1.3 Dynamic risk assessment

Complex systems thinking also facilitates more dynamic risk monitoring. In forensic contexts, early detection of shifts in risk state is essential. The network model, paired with monitoring, can support this. As discussed earlier, subtle changes (e.g., increased irritability or deteriorating sleep) can serve as early warning signs of destabilization (e.g., Kunkels et al., 2023). Empirical work has shown that markers of critical slowing, such as increased autocorrelation or variance, precede relapse in depression. Similarly, tracking changes in Mark’s personal network using, for example, ROM data could reveal increasing associations between variables like stress and anger, suggesting rising rigidity and heightened risk. Early identification of such shifts offers opportunities for timely intervention, such as time-outs, additional therapy, or medication adjustments, to stabilize the system before a crisis or recidivism occurs.

### 4.2 Future directions

#### 4.2.1 Communication and accessibility

Despite its promise, this integrative approach presents considerations. One concern is the complexity of communication. Network diagrams and transdiagnostic terminology can seem abstract to patients (and even to some professionals). Training and careful explanation are needed to convey why certain symptoms are prioritized and how they fit into the broader pattern. Nevertheless, visual tools like Figure 1 can aid understanding. When presented with guidance, many patients report appreciating the clarity a network map brings to their problems (Conway et al., 2023).

#### 4.2.2 Implementation in existing systems

Another topic is implementation within current care structures, which are often diagnosis centered. Introducing transdiagnostic interventions or network-informed diagnostics requires cultural and structural shifts (Levinson et al., 2023). For example, forensic clinics may organize care teams by disorder (e.g., addiction team, trauma team). A system-oriented model calls for multidisciplinary collaboration and flexibility, changes that are not trivial. Still, some institutions are already experimenting with process-based groups rather than disorder-based divisions, and with using ROM data to provide network feedback in therapy.

### 4.3 Scientific and methodological limitations

While each component, complex systems approach, transdiagnostic reasoning, and network modeling, has empirical support, research on their integration is still in its early stages. Future studies should evaluate whether the integrated approach, like the one used for Mark, leads to improved clinical outcomes. This call for outcome-based evaluation aligns with a growing movement in mental health research that emphasizes functional, cross-diagnostic interventions. A recent umbrella review (e.g., Solmi et al., 2025) found that physical exercise is an effective transdiagnostic intervention, producing moderate to strong benefits across conditions, such as depression, anxiety, PTSD, and substance use disorders. These findings highlight the value of targeting shared mechanisms like stress reactivity and cognitive control. Likewise, integrated network models must demonstrate that targeting central nodes, such as emotion regulation and impulsivity, can produce broad-spectrum clinical improvements, particularly in populations with complex, co-morbid psychopathology often encountered in forensic psychiatry. Key areas for exploration include the predictive value of networks for relapse or recidivism and the effectiveness of transdiagnostic treatment modules in forensic populations (e.g., Ebert et al., 2013). Methodologically, several open questions remain. How can we construct the most valid person-specific networks? Can we develop algorithms that distill key symptoms from routine clinical data and derive targeted interventions (e.g., Collin et al., 2022)? These questions reflect a deeper issue: how can we reliably understand the psychological traits and behaviors that form the building blocks of such networks? To move this approach forward, several developments are essential. First, person-specific networks require fine-grained, temporally rich data, ideally collected through EMA, wearable biosensors, and systematic behavioral observation. Such data allows us to conceptualize traits like impulsivity or mistrust as dynamic processes shaped by situational triggers (e.g., Bringmann et al., 2022; de Looff et al., 2024), rather than static characteristics. Second, new psychometric models are needed to transform these diverse data streams into clinically interpretable networks. For instance, emotion regulation issues might be captured not only through self-report but also through physiological responses, mood variability, and coping behaviors (de Looff et al., 2019). This enables behavior to be modeled as part of a dynamic, causal system. Third, machine learning methods offer new possibilities to identify core variables within individual networks. By analyzing patterns in both structured (e.g., routine outcome monitoring scores) and unstructured data (e.g., clinical notes), clinicians can more precisely determine which symptoms serve as central nodes influencing broader functioning (e.g., Berwian et al., 2025). These innovations are key to fulfilling the promise of network models: a shift from static diagnoses toward a personalized, dynamic understanding of mental health. This evolution is both a scientific challenge and a clinical imperative.

### 4.4 Ethical and legal considerations

The application of network models raises ethical and legal considerations. In the judicial context, questions arise regarding the extent to which these models are transparent and understandable to all parties involved. Clinicians must avoid overinterpreting statistical associations as deterministic evidence. Responsible implementation will require not only methodological rigor but also ethical awareness and interdisciplinary dialogue (Collin et al., 2022). These models are not intended for use in legal decision making, such as sentencing or individual risk prediction. They are designed to support clinical understanding and treatment planning.

### 4.5 Clinical implications for forensic practice

Despite these difficulties, the potential benefits of this approach are substantial. In the forensic setting, it can help clinicians navigate complex cases by clarifying priorities within a chaotic web of problems. Instead of reacting to incidents in isolation, teams can use network maps to determine where intervention will be most impactful. For instance, in Mark’s case, the team might prioritize sobriety as an immediate goal, since alcohol use exacerbates many other issues. At the same time, they might incorporate transdiagnostic skills training (e.g., from the Unified Protocol or DBT modules) to target improvements in emotion regulation and impulse control. The rationale for these choices is explicitly represented in the network, which can enhance both clinician and patient motivation by making the treatment logic transparent.

In forensic psychiatry specifically, this model moves risk management beyond static checklists. Traditional risk assessment tools (e.g., the HKT-R) include dynamic risk factors, but these are usually treated additively (Bogaerts et al., 2018). Recent longitudinal studies (e.g., Bogaerts et al., 2021; Bant and Bogaerts, 2024) show that the interaction and temporal evolution of these factors carry important information about treatment progress. The network model complements this by conceptualizing risk as an interactive system in which certain variables amplify one another. In Mark’s case, hostility/mistrust and impulsivity jointly increase the risk of violence, echoing findings that these traits are especially hazardous when co-occurring.

## 5 Conclusion

This paper presents a conceptual framework for understanding complex forensic psychopathology by integrating complexity theory, transdiagnostic processes, and network analysis. Rather than treating clinical problems as separate co-occurring diagnoses, this approach conceptualizes psychopathology as a dynamic system of interacting symptoms and underlying mechanisms. Applied to the case of Mark, it helps identify self-enforcing cycles and clinically relevant leverage points that are difficult to capture within traditional diagnostic models. This integrated conceptualization links theory with clinical reasoning and offers a starting point for more personalized care and coherent forensic practice. Mark’s case illustrates how combining complex systems thinking, transdiagnostic reasoning, and network analysis can move clinical decision making beyond fragmented diagnoses toward a more realistic understanding of interconnected problems, supporting both treatment coherence and public safety.

## Data Availability

The original contributions presented in the study are included in the article/supplementary material, further inquiries can be directed to the corresponding author.

## Footnotes

1 For a full case description and conceptual network models of Mark’s symptomatology and transdiagnostic processes, see “Case Description” in the Method section.
